# Supplementary material for: From sequence to enzyme mechanism using multi-label machine learning
Source: BMC Bioinformatics. 2014 May 19;15:150. doi: 10.1186/1471-2105-15-150 (PMC4229970; doi:10.1186/1471-2105-15-150)
Supplement: Additional file 2 — Java code of ml2db. Additional file ml2db_code.tar.gz contains the Java source code to run the multi-label machine learning experiments and save the results to database. The code’s Javadoc is included. [file 1471-2105-15-150-S2.zip › additional file 2/ml2db/ml2db/doc/mulan/data/LabelsBuilder.html]

LabelsBuilder


JavaScript is disabled on your browser.


- Overview
- Package
- Class
- Use
- Tree
- Deprecated
- Index
- Help

- Prev Class
- Next Class

- Frames
- No Frames

- All Classes

- Summary:
- Nested |
- Field |
- Constr |
- Method

- Detail:
- Field |
- Constr |
- Method


mulan.data

## Class LabelsBuilder

- java.lang.Object
- - mulan.data.LabelsBuilder

- ---

    

  ```
  public final class LabelsBuilder
  extends java.lang.Object
  ```

  The `LabelsBuilder` is responsible for creation of `LabelsMetaDataImpl` instance
  from specified XML file source. The builder ensures XML source validity against XML schema.

  Version:
  :   2012.02.26

  Author:
  :   Jozef Vilcek

- - ### Constructor Summary

    Constructors

    Constructor and Description

    `LabelsBuilder()`
  - ### Method Summary

    Methods

    | Modifier and Type | Method and Description |
    | --- | --- |
    | `static LabelsMetaData` | `createLabels(java.io.InputStream inputStream)` Creates a `LabelsMetaData` instance from specified input stream. |
    | `static LabelsMetaData` | `createLabels(java.lang.String xmlLabelsFilePath)` Creates a `LabelsMetaData` instance from XML file specified by the path. |
    | `static void` | `dumpLabels(LabelsMetaData labelsMetaData, java.io.OutputStream outputStream)` Dumps specified labels meta-data, in XML format, into the specified `OutputStream`. |
    | `static void` | `dumpLabels(LabelsMetaData labelsMetaData, java.lang.String xmlDumpFilePath)` Dumps specified labels meta-data into the file in XML format. |

    - ### Methods inherited from class java.lang.Object

      `equals, getClass, hashCode, notify, notifyAll, toString, wait, wait, wait`

- - ### Constructor Detail


    - #### LabelsBuilder

      ```
      public LabelsBuilder()
      ```
  - ### Method Detail


    - #### createLabels

      ```
      public static LabelsMetaData createLabels(java.lang.String xmlLabelsFilePath)
                                         throws LabelsBuilderException
      ```

      Creates a `LabelsMetaData` instance from XML file specified by the path.

      Parameters:
      :   `xmlLabelsFilePath` - the path to XML file containing labels definition

      Returns:
      :   the `LabelsMetaData` instance

      Throws:
      :   `ArgumentNullException` - if specified path to XML file is null
      :   `java.lang.IllegalArgumentException` - if file under specified path does not exists
      :   `LabelsBuilderException` - if specified file can not be read/opened


    - #### createLabels

      ```
      public static LabelsMetaData createLabels(java.io.InputStream inputStream)
                                         throws LabelsBuilderException
      ```

      Creates a `LabelsMetaData` instance from specified input stream.

      Parameters:
      :   `inputStream` - the input stream containing labels definition in XML format

      Returns:
      :   the `LabelsMetaData` instance

      Throws:
      :   `ArgumentNullException` - if specified input stream is null
      :   `LabelsBuilderException` - if any error occur when validating XML against
          schema or when creating labels data form specified input stream


    - #### dumpLabels

      ```
      public static void dumpLabels(LabelsMetaData labelsMetaData,
                    java.lang.String xmlDumpFilePath)
                             throws LabelsBuilderException
      ```

      Dumps specified labels meta-data into the file in XML format.
      If the file already exists, the content will be overwritten.

      Parameters:
      :   `labelsMetaData` - the meta-data which has to be dumped into the file
      :   `xmlDumpFilePath` - the path to the file where meta-data should be dumped

      Throws:
      :   `LabelsBuilderException` - if specified file can not be read/opened
      :   `ArgumentNullException` - if path to the file is not specified


    - #### dumpLabels

      ```
      public static void dumpLabels(LabelsMetaData labelsMetaData,
                    java.io.OutputStream outputStream)
                             throws LabelsBuilderException
      ```

      Dumps specified labels meta-data, in XML format, into the specified `OutputStream`.

      Parameters:
      :   `labelsMetaData` - the meta-data which has to be dumped
      :   `outputStream` - the output stream where XML dup will be written

      Throws:
      :   `LabelsBuilderException` - if error occurs during the serialization of meta-data to
          the XML format of resulting XML is not valid against the schema
      :   `ArgumentNullException` - if specified output strema is null.


- Overview
- Package
- Class
- Use
- Tree
- Deprecated
- Index
- Help

- Prev Class
- Next Class

- Frames
- No Frames

- All Classes

- Summary:
- Nested |
- Field |
- Constr |
- Method

- Detail:
- Field |
- Constr |
- Method
